# Deubiquitinase PSMD14 promotes ovarian cancer progression by decreasing enzymatic activity of PKM2

**DOI:** 10.1002/1878-0261.13076

**Published:** 2021-08-25

**Authors:** Tianshui Sun, Zhuonan Liu, Fangfang Bi, Qing Yang

**Affiliations:** ^1^ Department of Obstetrics and Gynecology Shengjing Hospital of China Medical University Shenyang China; ^2^ Department of Urology First Hospital of China Medical University Shenyang China; ^3^ Department of Obstetrics and Gynecology Shengjing Hospital of China Medical University Shenyang China

**Keywords:** cancer metabolism, deubiquitination, OPA, PKM2, PSMD14

## Abstract

Dysregulation of deubiquitination has been reported to contribute to carcinogenesis. However, the function and mechanism of deubiquitinating enzyme 26S proteasome non‐ATPase regulatory subunit 14 (PSMD14) in the progression of ovarian cancer (OV), the deadliest gynecological cancer, still remains to be characterized. The present study demonstrated that PSMD14 was overexpressed in OV tissues and its higher levels correlated with a higher International Federation of Gynecology and Obstetrics (FIGO) stage in OV patients. A high level of PSMD14 expression was related to poor survival in OV patients. Knockdown and overexpression experiments elucidated that PSMD14 stimulated OV cell proliferation, invasion, and migration *in vitro*. Repression of PSMD14 suppressed OV tumor growth *in vivo*. PSMD14 inhibitor O‐phenanthroline (OPA) effectively attenuated malignant behaviors of OV cells *in vitro* and OV tumor growth *in vivo*. Mechanistically, we uncovered that PSMD14 was involved in post‐translational regulation of pyruvate kinase M2 isoform (PKM2). PSMD14 decreased K63‐linked ubiquitination on PKM2, downregulated the ratio of PKM2 tetramers to dimers and monomers, and subsequently diminished pyruvate kinase activity and induced nuclear translocation of PKM2, contributing to aerobic glycolysis in OV cells. Collectively, our findings highlight the potential roles of PSMD14 as a biomarker and therapeutic candidate for OV.

## Graphical abstract

PSMD14 decreases K63‐linked ubiquitination on PKM2 and downregulates the ratio of PKM2 tetramers to dimers and monomers. Subsequently, this diminishes pyruvate kinase activity and induces nuclear translocation of PKM2 as well as transcription of downstream genes, contributing to aerobic glycolysis in ovarian cancer cells and progression of ovarian cancer.

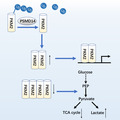

**Abbreviations**
CHX: cycloheximide
DUB: deubiquitinating enzyme
EMT: epithelial–mesenchymal transition
FBP: fructose 2,3‐bisphosphate
FIGO: Federation of Gynecology and Obstetrics
IHC: immunohistochemistry
JAMM: JAB1/MPN+/MOV34
OPA: O‐phenanthroline
OV: ovarian cancer
PK: pyruvate kinase
RT‐qPCR: reverse transcription‐quantitative polymerase chain reaction

## 1 Introduction

As the deadliest gynecological cancer and the fifth leading cause of cancer‐associated deaths in female, ovarian cancer caused 13 940 deaths in the United States in 2020 [1]. With lack of obvious symptoms and reliable detection methods at the early stage, about 70% of ovarian cancer patients are diagnosed at an advanced stage and have poor prognosis [2]. The 5‐year overall survival rate for ovarian cancer patients is < 45%, which has not been improved significantly in the past two decades [3]. Therefore, it is essential to clarify key mechanisms underlying ovarian cancer initiation and progression, with an aim to identify effective biomarkers for tumor diagnosis and to design novel therapies to improve prognosis.

Ubiquitination is a cascade process ligating ubiquitin to lysine residues of substrate proteins. This post‐translational modification is classified into monoubiquitination and polyubiquitination according to whether the attached monoubiquitin is modified further to produce a polyubiquitin chain [4]. Dependent on the attachment residue of ubiquitin, polyubiquitination is then divided into 8 types, including K6, K11, K27, K29, K33, K48, K63, and M1, leading to diverse fates of substrate proteins [5]. Deubiquitinating enzymes (DUBs) catalyze the process of removing ubiquitin from ubiquitinated substrates, which is called deubiquitination [6]. Because of the variety of substrates, deubiquitination plays important roles in multiple cellular functions [6, 7]. Correspondingly, dysregulation of deubiquitination has been reported to result in various diseases, including cancers [6, 7].

As a subunit of 19S regulatory particle in 26S proteasome, 26S proteasome non‐ATPase regulatory subunit 14 (PSMD14, also known as RPN11 and POH1), belongs to the JAB1/MPN+/MOV34 (JAMM) domain protease family of DUBs and has been reported to be involved in carcinogenesis and progression of several human cancers [8, 9, 10]. Previous studies have demonstrated that PSMD14 is upregulated in cancers, such as prostate cancer and hepatocellular carcinoma [8, 9, 11, 12, 13, 14]. Excessive expression of PSMD14 is associated with higher TNM stage in lung adenocarcinoma, increased vascular invasion in hepatocellular carcinoma, higher pathological grade in prostate cancer, and poor survival in patients with colorectal cancer or breast cancer [8, 9, 11, 13, 14, 15]. Downregulation of PSMD14 expression can induce G0/G1 phase arrest, apoptosis, and can diminish proliferation and epithelial–mesenchymal transition (EMT) in lung adenocarcinoma, prostate cancer, and breast cancer cells [9, 11, 13, 14, 16]. Mechanistically, PSMD14 has been observed to play oncogenic roles in head and neck squamous cell carcinoma, glioma, and hepatocellular carcinoma by inhibiting ubiquitination and degradation of cancer‐associated transcriptional factor E2F1 [8, 17, 18]. PSMD14 can also mediate deubiquitination and enhance the stability of ALK2, an oncogenic serine/threonine kinase, thereby promoting tumor growth and chemoresistance in colorectal cancer [15]. Increased stability of GRB2 and TGF‐β receptors induced by PSMD14 overexpression can hyperactivate their signaling to enhance hepatocellular carcinoma growth and metastasis [9, 19]. PSMD14 also promotes EMT in esophageal squamous cell carcinoma by targeting SNAIL for deubiquitination and stabilization [20]. Therefore, PSMD14 acts as an oncogene in several cancers by deubiquitinating diverse protein substrates. However, the function and mechanism of PSMD14 in ovarian cancer still need to be investigated.

In the present study, we firstly reported the oncogenic roles for PSMD14 in ovarian cancer by detecting its clinical significance in ovarian cancer patients and exploring its influence on malignant behaviors of ovarian cancer *in vitro* and *in vivo*. Mechanistically, we observed that it regulated M2 isoform of pyruvate kinase (PKM2) post‐translationally. PSMD14‐mediated deubiquitination of PKM2 induces decreased ratio of PKM2 tetramer compared with PKM2 dimer and monomer, diminishing PK activity to induce aerobic glycolysis and promoting nuclear translocation of PKM2 to enhance the transcription of downstream cancer‐promoting genes, which thereby stimulates malignant progression of ovarian cancer.

## 2 Materials and methods

### 2.1 Patients and tissue samples

Ovarian primary cancer and normal ovarian tissues were obtained from patients who had undergone resection in Shengjing Hospital of China Medical University from 2014 to 2017. The pathological diagnosis was confirmed by two clinical pathologists. None of the ovarian cancer patients had received chemotherapy before the surgery, and all the patients had received standardized cytoreductive surgery and postoperative chemotherapy at Shengjing Hospital of China Medical University. Ovarian cancer patients were followed through August 30, 2018. The study was approved by the Research Ethics Committee of China Medical University, and all patients signed written informed consent. Our study methodologies conformed to the standards set by the Declaration of Helsinki.

### 2.2 Immunohistochemistry (IHC)

After fixation of tissues with 10% neutral formalin, 4‐μm‐thick paraffin‐embedded tissue sections were prepared. Following deparaffinized, hydrated, and soaked in 3% H2O2 for 20 min at room temperature, the sections were incubated with anti‐PSMD14 (1 : 500, ab109123, Abcam, Cambridge, UK) antibodies at 4 °C overnight. The slides were incubated with biotinylated goat anti‐rabbit antibodies for 1 h, stained with diaminobenzidine (DAB; Maixin Biotechnology, Fuzhou, China), and then counterstained with hematoxylin (Maixin Biotechnology). The IHC sections were assessed by two independent pathologists blinded to the experimental data. The specimen was scored according to the percentage of positive staining cells (0 = negative; 1 = 1–10%; 2 = 11–50%; 3 = 51–90%; 4 = 91–100%) and the intensity of staining (0 = no staining; 1 = slight staining; 2 = moderate staining; 3 = strong staining). Scores for the percentage and intensity of staining were added. The score of 0–3 was considered as a low‐expression group, and the score of 4–7 was considered as a high‐expression group.

### 2.3 Cell culture, lentiviral infection, and transfection

A2780, OVCAR‐3, and HO‐8910 cells were purchased from the Chinese Academy of Sciences Cell Bank (Shanghai, China) and were cultured in RPMI 1640 medium (Bioind, Kibbutz Beit Haemek, Israel) supplemented with 10% fetal bovine serum (FBS; Bioind). HEK293T cell were acquired from the American Type Culture Collection (ATCC, Manassas, VA, USA) and were cultured in high glucose Dulbecco’s modified Eagle medium (DMEM, Bioind) supplemented with 10% FBS. The cell lines were authenticated by comparing with the STR database. Cells were cultured at 37 °C in a 5% CO2 atmosphere and were detected regularly by using PCR for mycoplasma contamination.

The lentivirus that expresses PSMD14 shRNAs and the negative control vectors were ordered from GeneChem (Shanghai, China). The A2780, OVCAR‐3, and HO‐8910 cells were infected with lentivirus at the multiplicity of infection (MOI) of 10, 30, and 10 for 48 h, respectively. The plasmids that express Flag‐PSMD14, Myc‐PKM2, or HA‐ubiquitin were purchased from GeneChem. Small interfering RNAs (siRNAs) targeting PKM2 were purchased from GenePharma (Suzhou, China). The sequences for siRNAs are shown in Table S1. Lipofectamine 3000 (Invitrogen, Carlsbad, CA, USA) was used for transfection according to the manufacture’s protocol.

### 2.4 Cell viability assay

CCK‐8 kit (Bimake, Houston, TX, USA) was used to evaluate cell viability. Cell suspension was inoculated in 96‐well plates (3 × 10^3^ cells per well). CCK‐8 solution (10 μL) was added to the wells every 24 h. After incubation for 1.5 h, the absorbance was measured at 450 nm using a microplate reader.

### 2.5 Colony formation assay and soft agarose assay

For colony formation assays, cells were plated into 6‐well plates (400 cells per well). After cultured for 2 weeks, cells were fixed with 4% paraformaldehyde and stained with 0.5% crystal violet. Colonies (> 50 cells) were manually counted.

For soft agarose assay, we prepared two different concentrations (20 and 6 g·L^−1^) of low‐melting point agarose gels (Solarbio, Beijing, China). After sterilization, gels were melted and maintained at 40 °C. 1 mL 1% soft agarose gel was obtained by mixing 500 μL 2×RPMI 1640 medium with 500 μL 20 g·L^−1^ agarose gel and was then added into well of 6‐well plate. After the layer of 1% soft agarose gel was clotted at 4 °C, 1 mL 0.3% soft agarose gel was prepared by mixing 500 μL 2×RPMI 1640 medium containing 4 × 10^3^ cells with 500 μL 6 g·L^−1^ agarose gel and was added into the well to form the upper layer. After the cells were cultured for 2 weeks, microscope was used for observation of colony formation.

### 2.6 Transwell assay

Matrigel invasion assays were performed in 24‐well plates using Transwell polycarbonate filters with an 8.0‐μm pore size (Corning, New York, NY, USA). 600 μL medium containing 10% FBS was added to the lower chamber. Cells in serum‐free medium (2 × 10^4^ cells/200 μL) were seeded into the upper chambers precoated with Matrigel (BD Biosciences, San Jose, CA, USA) and were incubated at 37 °C for 24 h. Then, cells were fixed with 4% paraformaldehyde and stained with 0.5% crystal violet. The invaded cells were photographed and counted.

### 2.7 Cell scratch assay

Single cell suspensions were seeded into 6‐well plates. After 24 h, the plate was gently scratched in a straight line with a 200‐μL pipette tip and washed with PBS and then cultured in medium without serum for 24 h. The scratch width was measured under microscope at 0 and 24 h.

### 2.8 Reverse transcription‐quantitative polymerase chain reaction (RT‐qPCR)

Total RNA was extracted using the RNAiso Plus reagent (Takara Bio, Kusatsu, Japan), assessed with a NanoDrop 2000 system (Thermo Fisher, Carlsbad, CA, USA) for RNA concentration and purity, and then reverse transcribed using the PrimeScript™ RT reagent Kit with gDNA Eraser (Perfect Real Time) (Takara Bio). Real‐time quantitative polymerase chain reaction was performed using SYBR® Premix Ex Taq™ II (Tli RNaseH Plus) (Takara Bio) on an ABI 7500 Fast system (Life Technologies, Carlsbad, CA, USA). The primer sequences were listed in Table S1. Gene expression was calculated relative to that of ACTB using the $2^{‐\Delta \Delta C_{t}}$ method.

### 2.9 Western blot

Total cell proteins were extracted by using RIPA lysate (Beyotime, Shanghai, China). Subcellular fractionation was performed by using a Nuclear and Cytoplasmic Protein Extraction kit (Beyotime) according to the manufacturer’s instructions. Proteins were separated by using 7.5% or 10% SDS/PAGE and transferred onto PVDF membranes (Millipore, Temecula, CA, USA). The membranes were blocked for 2 h at room temperature and were incubated with primary anti‐PSMD14 (1 : 1000, 4197, Cell Signaling Technology, Danvers, MA, USA), anti‐beta‐actin (1 : 1000, 3700, Cell Signaling Technology), anti‐Flag tag (1 : 1000, 66008‐3‐Ig, ProteinTech, Wuhan, Hubei, China), anti‐Myc tag (1 : 1000, 16286‐1‐AP, ProteinTech), anti‐PKM2 (1 : 1000, 15822‐1‐AP, ProteinTech), anti‐PKM1 (1 : 1000, 15821‐1‐AP, ProteinTech), anti‐ubiquitin (1 : 1000, 3936, Cell Signaling Technology), anti‐HA tag (1 : 1000, 3724, Cell Signaling Technology), or anti‐Histone H3 (1 : 1000, 4499, Cell Signaling Technology) antibodies. The membranes were then washed and incubated with secondary antibodies. Protein bands were detected by using enhanced chemiluminescence (Thermo Scientific, Carlsbad, CA, USA).

### 2.10 Animal study

A xenograft mouse model was constructed using 5‐week‐old female BALB/c nude mice (Beijing HFK Bioscience, Changping, Beijing, China). All mice were given free access to food and water and were fed in an SPF (specific pathogen‐free) animal facility. A total of 2 × 10^7^ cells resuspended in 200 μL of PBS was injected subcutaneously into the left axilla of the nude mice. Tumor size was measured every 3 days using a Vernier caliper, and tumor volumes were calculated using the formula *V* = 1/2(length × width^2^). These mice were sacrificed 15 days after injection. All animal experiments were approved by the Animal Care and Use Committee of China Medical University.

### 2.11 Mass spectrometry

The OVCAR‐3 cells were lysed after incubation with 10 μm MG132 for 6 h. The cell lysates were immunoprecipitated with anti‐PSMD14 antibodies (4197, Cell Signaling Technology) or negative control IgG (2729, Cell Signaling Technology) overnight and subsequently conjugated with Dynabeads Protein (10001D, Invitrogen) for an additional incubation of 2 h. Gel electrophoresis was run to prepare samples for subsequent proteomic analysis. The PAGE Gel Silver Staining Kit (Solarbio) was used to perform silver staining of the gel. The LC‐MS/MS analysis of the samples to identify proteins interacting with PSMD14 was performed by Novogene (Beijing, China). The Co‐IP gel bands and their corresponding negative gel bands were excised and digested using in‐gel trypsin. The extracted peptide mixtures were analyzed using an EASY‐nLCTM 1200 UHPLC system (Thermo Fisher) coupled with an Q Exactive HF‐X mass spectrometer (Thermo Fisher) with ion source of Nanospray Flex™ electrospray ionization (Thermo Fisher). The spectra from each peptide were matched separately against UNIPROT human protein database by using Proteome Discoverer 2.2 (PD 2.2, Thermo). The search parameters were set as follows: Mass tolerance for precursor ion was 10 ppm, and mass tolerance for product ion was 0.02 Da; carbamidomethyl was specified in PD 2.2 as fixed modifications; oxidation of methionine (M) and acetylation of the N‐terminus were specified in PD 2.2 as variable modifications; and a maximum of 2 missed cleavage sites was allowed. The proteins containing at least 1 unique peptide with FDR no more than 1.0% were selected. The mass spectrometry proteomic data have been deposited to the ProteomeXchange Consortium via the Proteomics Identifications (PRIDE) partner repository with the dataset identifier PXD027016.

### 2.12 Co‐immunoprecipitation (Co‐IP) assay

Exogenous Co‐IP was performed after HEK293T cells were transfected with indicated plasmids for 48 h. After incubation with 10 μm MG132 for 6 h, cells in 10‐cm dish were lysed in co‐immunoprecipitation lysis buffer (Beyotime) with proteinase inhibitors. Cell lysates were immunoprecipitated with anti‐PSMD14 (1 : 100; 4197; Cell Signaling Technology), anti‐Flag tag (1 μg/100 μL; 66008‐3‐Ig; ProteinTech), anti‐Myc tag (1 μg/100 μL; 16286‐1‐AP; ProteinTech), anti‐PKM2 (1 μg/100 μL; 15822‐1‐AP; ProteinTech) antibodies, or negative control IgG (1 : 100; 2729; Cell Signaling Technology) at 4 °C overnight. Then, 50 μL Dynabeads was added into the lysates and incubated with rotation at 4 °C for 2 h. The tube was placed on the magnet, and the supernatant was removed. The Dynabeads–antibody–antigen complexes were washed for five times gently using 500 μL of washing buffer. The tube was placed on the magnet, and the supernatant was removed after each wash. Dynabeads–antibody–antigen complexes were then gently resuspended in SDS sample buffer and denatured by heating for 20 min at 100 °C. The tube was placed on the magnet, and the supernatant was collected for western blot analyses.

### 2.13 Cross‐linking assays

Cells were lysed in sodium phosphate buffer (pH 7.3) containing 0.5% Triton X‐100 and protease inhibitor for 30 min at 4 °C. After centrifugation of cell lysates at 26 600 ***g*** for 30 min at 4 °C, the supernatants were collected and treated with 0.02% glutaraldehyde for 5 min at 37 °C. 50 mm Tris‐Cl (pH 8.0) was used to terminate the reactions. The samples were then collected for western blot analyses.

### 2.14 Pyruvate kinase (PK) activity, glucose uptake, and lactate production assay

PK activities in the cellular extracts were measured with a PK Activity Colorimetric Assay Kit according to the manufacturer’s instructions (K709‐100, BioVision, Milpitas, CA, USA). The amount of glucose uptake was measured with a Colorimetric Glucose Uptake Assay Kit according to the manufacturer’s protocol (36504, AAT Bioquest, Sunnyvale, CA, USA). The lactate production was measured using a Lactate Colorimetric Assay Kit II (K627‐100, BioVision) according to the manufacturer’s instructions.

### 2.15 Immunofluorescence staining

After fixation with 4% paraformaldehyde for 30 min and permeabilization with 0.5% Triton X‐100 for 10 min, cells were gently washed with PBS and were blocked with goat serum for 40 min and then were incubated with anti‐Flag tag (1 : 100, 66008‐3‐Ig, ProteinTech), anti‐Myc tag (1 : 100, 16286‐1‐AP, ProteinTech), or anti‐PKM2 (1 : 100, 15822‐1‐AP, ProteinTech) antibodies diluted in blocking buffer overnight at 4 °C. After gently washed five times for 5 min each with PBS, cells were then incubated with the corresponding fluorescence‐conjugated secondary antibodies for 2 h at room temperature. Then, the cells were gently washed with PBS, and nuclei were stained with DAPI for 10 min. The cells were washed again, and the coverslips were mounted by fluorescence decay‐resistant mounting medium onto slides. The cells were photographed by using a confocal laser‐scanning microscope (Nikon,Tokyo, Japan).

### 2.16 Statistical analysis

Statistical analyses were performed with graphpad prism 7.0 (GraphPad Software, San Diego, CA, USA). Data were expressed as the mean ± standard deviation of at least three independent experiments. The count data and the measurement data were analyzed using chi‐squared tests and *t*‐test, respectively. Survival analysis was performed by the Kaplan–Meier method and the log‐rank test. *P* < 0.05 was considered statistically significant. *P < 0.05; **P < 0.01; ***P < 0.001.

## 3 Results

### 3.1 PSMD14 is overexpressed in ovarian cancer and associated with ovarian cancer progression

**Fig. 1:**
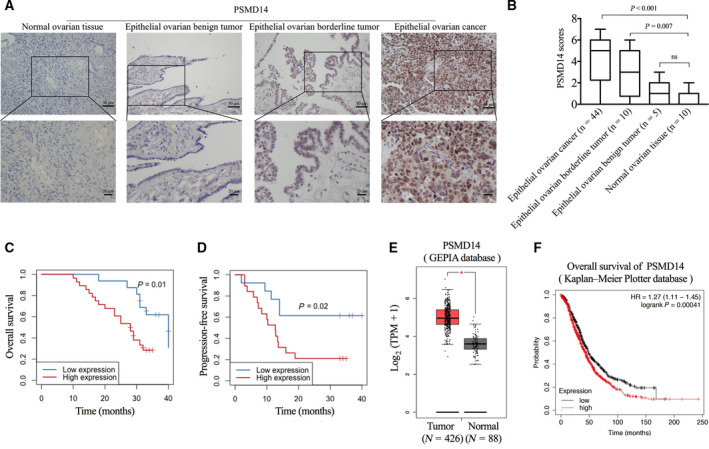
PSMD14 is overexpressed in ovarian cancer tissues, and high level of PSMD14 expression is associated with poor prognosis in ovarian cancer patients. (A) Representative images of immunohistochemical staining of PSMD14 in epithelial ovarian cancer (*n* = 44), epithelial ovarian borderline tumor (*n* = 10), epithelial ovarian benign tumor (*n* = 5), and normal ovarian tissues (*n* = 10). Scale bars, 50 μm; 20 μm. (B) Differences in PSMD14 scores in epithelial ovarian cancer (*n* = 44), epithelial ovarian borderline tumor (*n* = 10), epithelial ovarian benign tumor (*n* = 5), and normal ovarian tissues (*n* = 10) are presented as a box plot. (C) Kaplan–Meier plots for the overall survival of ovarian cancer patients in PSMD14 low (*n* = 16) and high (*n* = 28) groups. (D) Kaplan–Meier plots for the progression‐free survival of ovarian cancer patients in PSMD14 low (*n* = 16) and high (*n* = 28) groups. (E) mRNA expression levels of PSMD14 in ovarian cancer and normal ovarian tissues from the GEPIA database. (F) Kaplan–Meier plots for the overall survival of ovarian cancer patients according to PSMD14 mRNA expression from the Kaplan–Meier Plotter database. Error bars in B and E indicated mean ± SD. Data analysis in B and E was conducted by unpaired *t*‐test. Survival analyses in C, D, and F were performed by the Kaplan–Meier method and the log‐rank test. *P < 0.05.

To explore the expression of PSMD14 in ovarian cancer, we first investigated the expression of PSMD14 in 44 primary epithelial ovarian cancer specimens, 10 epithelial ovarian borderline tumor, 5 epithelial ovarian benign tumor, and 10 normal ovarian tissues via IHC assay. The median ages of the primary epithelial ovarian cancer group, the epithelial ovarian borderline tumor group, the epithelial ovarian benign tumor group, and the normal ovarian tissue group were 54 years (range 36–75), 51 years (range 29–71), 47 years (range 26–63), and 56 years (range 48–62), respectively. There was no significant difference between the ages of different groups. We observed higher PSMD14 level in primary epithelial ovarian cancer and epithelial ovarian borderline tumor compared with that in normal ovarian tissues (Fig. 1A,B). PSMD14 was highly expressed in 64% of primary epithelial ovarian cancer specimens, 50% of epithelial ovarian borderline tumor specimens, and none of the epithelial ovarian benign tumor or normal ovarian tissues. Statistical analyses showed that upregulated PSMD14 expression was positively correlated with higher International Federation of Gynecology and Obstetrics (FIGO) stage (Table 1), indicating that overexpressed PSMD14 contributes to ovarian cancer progression. Kaplan–Meier analyses revealed that high PSMD14 expression was related to poor overall survival and progression‐free survival in ovarian cancer patients (Fig. 1C,D). The median overall survival time in the low‐expression group was 40 months, while that in the high‐expression group was 28 months. To verify these results, we further used GEPIA database and consistently observed higher expression level of PSMD14 in ovarian cancer tissues compared with that in normal ovarian tissues (Fig. 1E). In the Kaplan–Meier plotter database, the level of PSMD14 expression was found to be negatively associated with overall survival as well (Fig. 1F). Taken together, these data suggest that upregulated PSMD14 is associated with ovarian cancer progression.

**Table 1:** Relationships between PSMD14 expression in epithelial ovarian cancer and clinicopathological parameters.

Characteristic	*n*	Low	High	*P* value
Age (years)				
≤ 55	24	9	15	0.864
> 55	20	7	13	
Stage				
FIGO I/II	9	7	2	0.004
FIGO III/IV	35	9	26	
Grade				
Well/moderate	15	8	7	0.092
Poor	29	8	21	
Pathologic type				
Serous	38	14	24	0.832
Mucinous	1	0	1	
Endometrioid	2	1	1	
Clear cell carcinoma	2	1	1	
Others	1	0	1	

FIGO, International Federation of Gynecology and Obstetrics.

### 3.2 PSMD14 promotes proliferation, invasion, and migration of ovarian cancer cells *in vitro* and ovarian tumor growth *in vivo*

**Fig. 2:**
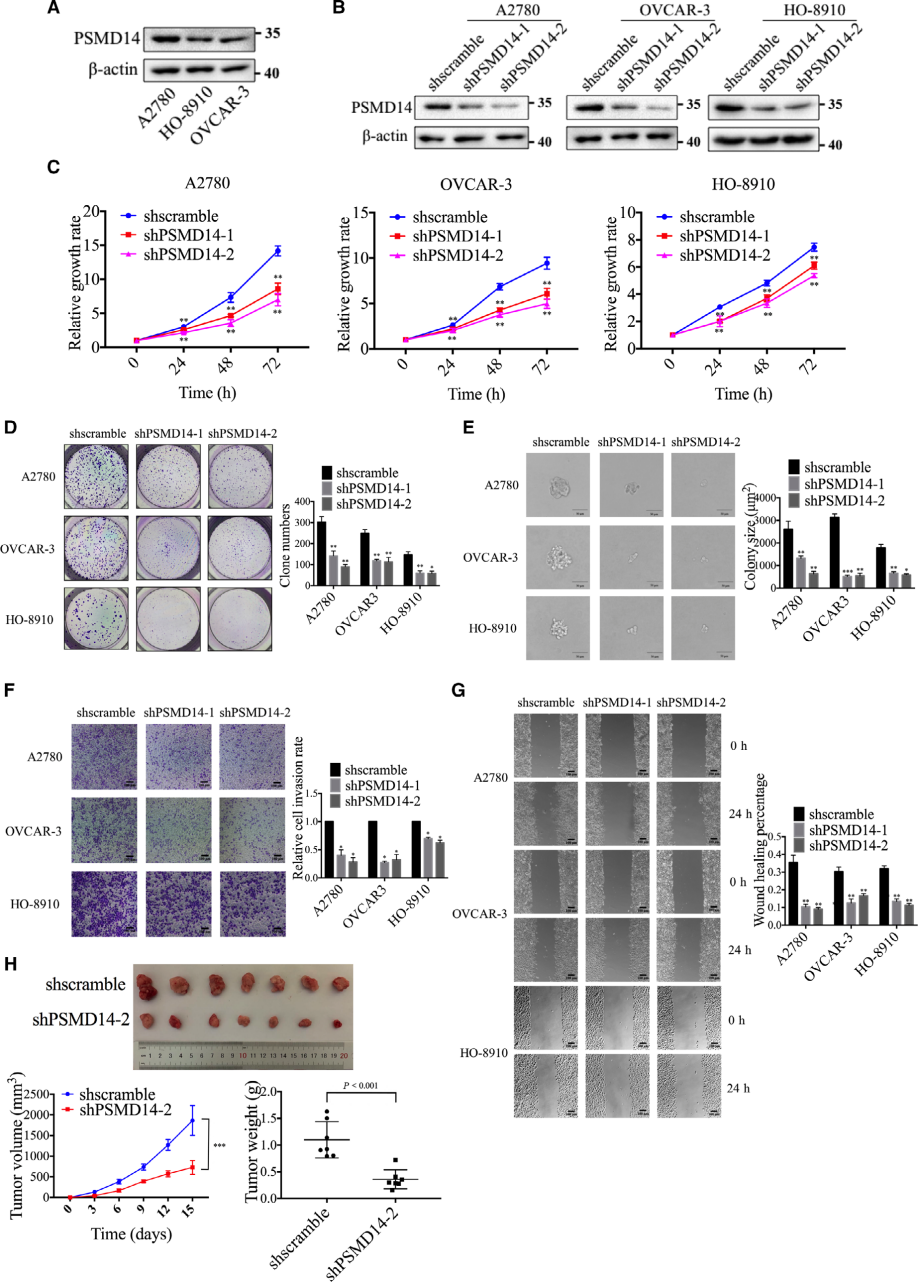
PSMD14 knockdown inhibits proliferation, invasion, and migration of ovarian cancer cells *in vitro* and ovarian tumor growth *in vivo*. (A) Endogenous expression levels of PSMD14 protein in A2780, HO‐8910, and OVCAR‐3 ovarian cancer cell lines were detected by western blot. (B) Efficiency of PSMD14 knockdown in A2780, OVCAR‐3, and HO‐8910 cell lines was examined by western blot. (C) Cell viability was detected by CCK‐8 assays after PSMD14 knockdown. (D) Colony formation assay was used to detect the proliferation of ovarian cancer cells after knockdown of PSMD14. (E) Soft agarose assay was used for the quantification of anchorage‐independent growth capacity of ovarian cancer cells after knockdown of PSMD14. Scale bar, 50 μm. (F) Cell invasion was detected by Transwell assay after PSMD14 knockdown. Scale bar, 100 μm. (G) Cell scratch assay was used to analyze cell migration after PSMD14 knockdown. Scale bar, 100 μm. (H) Scramble control or PSMD14 knockdown OVCAR‐3 cells were subcutaneously injected into female nude mice for the observation of tumor growth. The tumor weight of xenograft from each group (*n* = 7) was calculated. Error bars in C–H indicated mean ± SD. Data analyses in C–H were conducted by unpaired *t*‐test. *P < 0.05; **P < 0.01; ***P < 0.001. Data represent three independent sets of experiment.

To investigate the functions of PSMD14 in the malignant biological behaviors of ovarian cancer cells, we firstly examined the endogenous expression of PSMD14 in three ovarian cancer cell lines, including A2780, HO‐8910, and OVCAR‐3 (Fig. 2A). We then downregulated PSMD14 expression by using shRNAs in these cells (Fig. 2B). CCK‐8 experiment results revealed that underexpressed PSMD14 reduced cell viability (Fig. 2C). Downregulation of PSMD14 also inhibited colony formation in ovarian cancer cells (Fig. 2D,E). Via Transwell and scratch assays, invasion and migration abilities of cells were demonstrated to be significantly attenuated after PSMD14 repression (Fig. 2F,G). To further explore the role of PSMD14 in ovarian cancer growth *in vivo*, female BALB/c nude mice were subcutaneously injected with the control and PSMD14 stable knockdown OVCAR‐3 cells. *In vivo* growth was impaired in tumor formed by PSMD14 stable knockdown cells compared with tumor formed by the control cells (Fig. 2H). In addition, PSMD14 stable knockdown also contributed to significantly lower ovarian tumor weight (Fig. 2H).

**Fig. 3:**
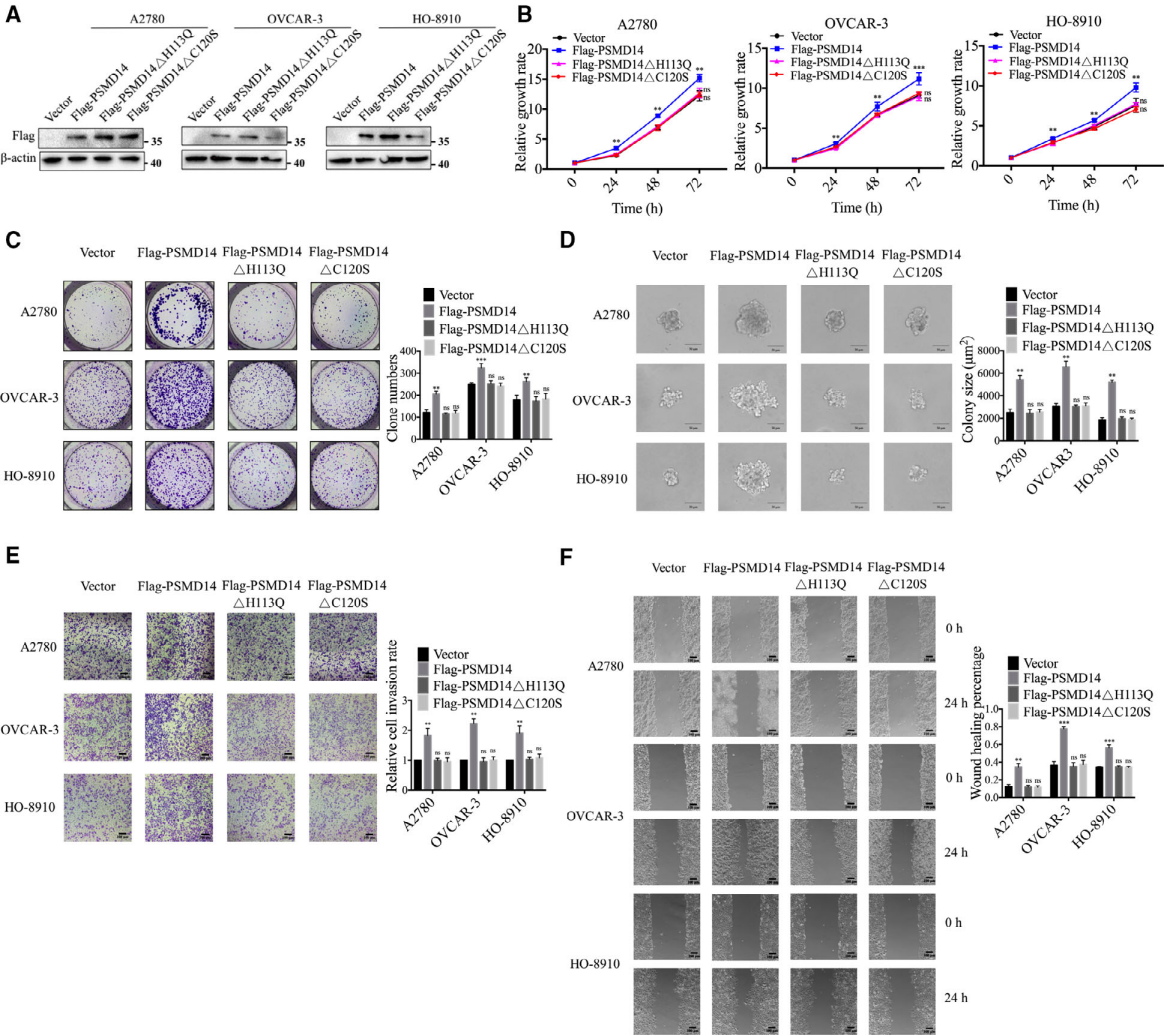
PSMD14 overexpression enhances proliferation, invasion, and migration of ovarian cancer cells *in vitro*. (A) Efficiency of PSMD14, PSMD14^▵H113Q^, and PSMD14^▵C120S^ overexpression in A2780, OVCAR‐3, and HO‐8910 cells was examined by western blot. (B) Cell viability was detected by CCK‐8 assays after overexpression of PSMD14 or PSMD14 mutants. (C) Colony formation assay was used to detect the proliferation of ovarian cancer cells after overexpression of PSMD14 or PSMD14 mutants. (D) Soft agarose assay was used for the quantification of anchorage‐independent growth capacity of ovarian cancer cells after overexpression of PSMD14 or PSMD14 mutants. Scale bar, 50 μm. (E) Cell invasion was detected by Transwell assay after overexpression of PSMD14 or PSMD14 mutants. Scale bar, 100 μm. (F) Cell scratch assay was used to analyze cell migration after overexpression of PSMD14 or PSMD14 mutants. Scale bar, 100 μm. Error bars in B–F indicated mean ± SD. Data analyses in B–F were conducted by unpaired *t*‐test. *P < 0.05; **P < 0.01; ***P < 0.001. Data represent three independent sets of experiment.

PSMD14 is a DUB, and according to previous studies, its H113 and C120 sites are crucial for its DUB activity [17]. Subsequently, we established ovarian cancer cell lines stably overexpressing wild‐type PSMD14 or PSMD14 mutants H113Q or C120S to investigate whether PSMD14 exerted oncogenic functions in ovarian cancer via its DUB activity (Fig. 3A). Overexpressed wild‐type PSMD14, but not PSMD14 mutants, was observed to induce promotion on cell viability and colony formation in ovarian cancer cells (Fig. 3B–D). According to the results of Transwell and scratch assays, the invasion and migration abilities of ovarian cancer cells elevated after upregulation of wild‐type PSMD14, but did not change significantly after overexpression of PSMD14 mutants (Fig. 3E,F). Collectively, PSMD14 plays oncogenic roles in ovarian cancer by stimulating proliferation, invasion, and migration, and its DUB activity is essential for its functions in ovarian cancer progression.

### 3.3 PSMD14 inhibitor OPA effectively represses the malignant behavior of ovarian cancer

**Fig. 4:**
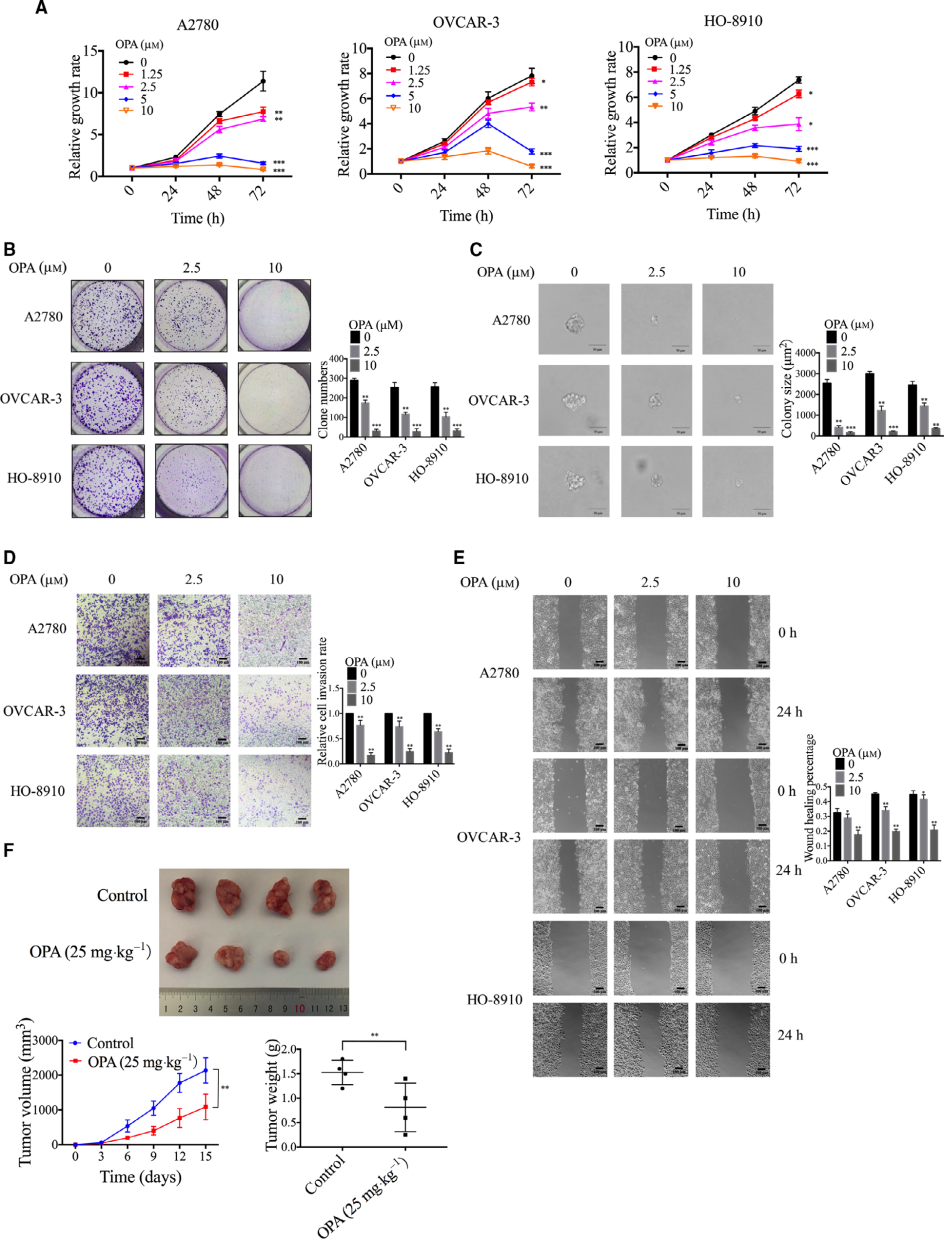
PSMD14 inhibitor O‐phenanthroline (OPA) inhibits the malignant behavior of ovarian cancer cells *in vitro* and ovarian tumor growth *in vivo*. (A) The cytotoxicity of OPA to ovarian cancer cells was examined by CCK‐8 assay. (B) The effect of OPA on proliferation of ovarian cancer cells was determined by colony formation assay. (C) After pretreated with OPA for 24 h, the effect of OPA on anchorage‐independent growth capacity of ovarian cancer cells was determined by soft agarose assay. Scale bar, 50 μm. (D) After pretreated with OPA for 24 h, ovarian cancer cells were then analyzed by Transwell assay to examine their invasion ability. Scale bar, 100 μm. (E) After pretreated with OPA for 24 h, ovarian cancer cells were then investigated by cell scratch assay to measure their migration ability. Scale bar, 100 μm. (F) Female BALB/c nude mice were treated with vehicle control or OPA (25 mg·kg^−1^) intraperitoneally every 3 days after subcutaneous injection of OVCAR‐3 cells. The tumor weight of xenograft from each group (*n* = 4) was calculated. Error bars in this figure indicated mean ± SD. Data analyses in this figure were conducted by unpaired *t*‐test. *P < 0.05; **P < 0.01; ***P < 0.001. Data represent three independent sets of experiment.

To investigate the therapeutic effect of PSMD14 inhibition on ovarian cancer, we next treated A2780, OVCAR‐3, and HO‐8910 cells with a reported PSMD14 inhibiter named O‐phenanthroline (OPA) [12]. After treating cells with different concentrations of OPA, we observed that OPA reduced viability of ovarian cancer cells in a concentration‐dependent manner (Fig. 4A). Treatment of OPA also inhibited colony formation in ovarian cancer cells (Fig. 4B,C). Furthermore, compared to DMSO‐treated cells, ovarian cancer cells treated with OPA markedly reduced their ability of migration and invasion (Fig. 4D,E). Moreover, to analyze the effect of OPA on ovarian tumor growth *in vivo*, female BALB/c nude mice were treated with OPA intraperitoneally every 3 days after subcutaneous injection of OVCAR‐3 cells. Results indicated that treatment of OPA inhibited ovarian tumor growth *in vivo* (Fig. 4F). To confirm the specificity of OPA against PSMD14 protein, we further treated PSMD14 stable knockdown ovarian cancer cells with OPA. But we did not observe pronounced reduction in the growth, invasion, or migration of these cells (Fig. S1), demonstrating that OPA impaired the malignant behavior of ovarian cancer cells by specifically targeting PSMD14. Taken together, these data demonstrated an inhibitory effect of PSMD14 inhibitor OPA on ovarian cancer progression.

### 3.4 PSMD14 interacts with PKM2 and decreases ubiquitination on PKM2

**Fig. 5:**
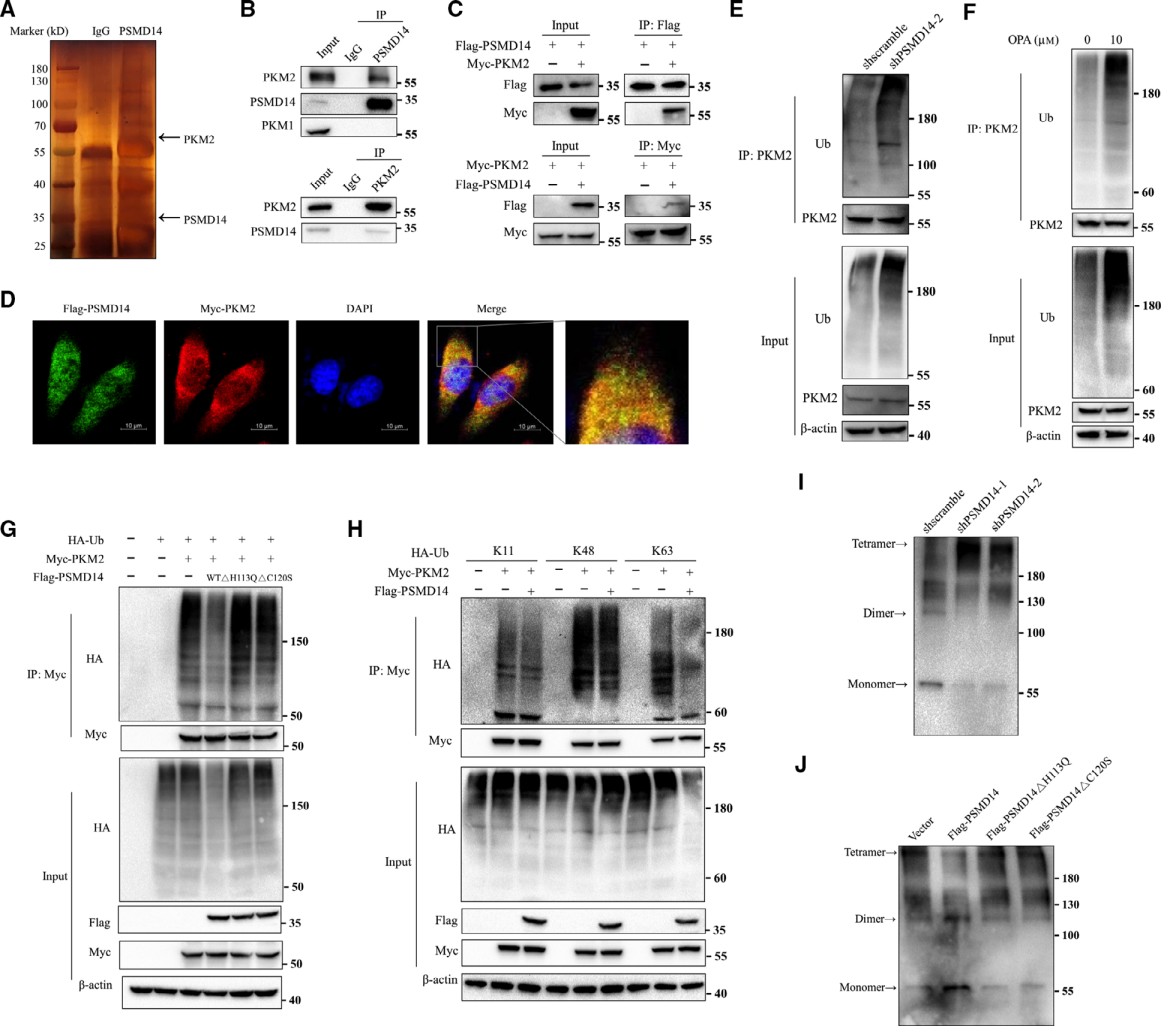
PSMD14 mediates deubiquitination of PKM2 and downregulates the ratio of PKM2 tetramer compared with PKM2 dimer and monomer. (A) Proteins that interacted with PSMD14 were identified by combining Co‐IP and mass spectrometry methods. (B) OVCAR‐3 cell lysates were immunoprecipitated with anti‐PSMD14 and anti‐PKM2 antibodies, respectively, and then analyzed by immunoblotting. The IgG antibody was used as control. (C) The Flag‐PSMD14 and Myc‐PKM2 plasmids were co‐transfected into HEK293T cells. Cell lysates were then immunoprecipitated by the anti‐Flag or anti‐Myc antibodies, respectively, and analyzed by immunoblotting. (D) After co‐transfected with Flag‐PSMD14 and Myc‐PKM2 plasmids, HEK293T cells were analyzed by immunofluorescence for detecting the localization of PSMD14 (green) and PKM2 (red). DAPI was used as a nuclear stain (blue). Scale bar, 10 μm. (E) Cell lysates of control and PSMD14 knockdown OVCAR‐3 cells were immunoprecipitated with anti‐PKM2 antibody and then were analyzed by immunoblotting with anti‐PKM2 and anti‐ubiquitin antibodies. (F) After treated with OPA for 24 h, cell lysates of OVCAR‐3 cells were immunoprecipitated with anti‐PKM2 antibody and then were analyzed by immunoblotting with anti‐PKM2 and anti‐ubiquitin antibodies. (G) Cell lysates of HEK293T cells overexpressing Flag‐PSMD14 or Flag‐PSMD14 mutants, Myc‐PKM2, and HA‐ubiquitin were immunoprecipitated with anti‐Myc antibody and then detected by western blot analysis with anti‐HA and anti‐Myc antibodies. (H) Cell lysates of HEK293T cells overexpressing Flag‐PSMD14, Myc‐PKM2, and different forms of HA‐ubiquitin were immunoprecipitated with anti‐Myc antibody and then detected by western blot analysis with anti‐HA and anti‐Myc antibodies. (I) Cell lysates of control and PSMD14 knockdown OVCAR‐3 cells were treated by cross‐linking to detect PKM2 tetramers, dimers, and monomers with PKM2 antibody. (J) Cell lysates of control and PSMD14 or PSMD14 mutants overexpressed OVCAR‐3 cells were treated by cross‐linking to detect PKM2 tetramers, dimers, and monomers with PKM2 antibody. Data represent three independent sets of experiment.

To disclose the mechanism of PSMD14 in promoting ovarian cancer progression, a mass spectrometry analysis was carried out (Fig. 5A), and 406 proteins were identified to be co‐immunoprecipitated with PSMD14 in ovarian cancer cells (Table S2). Among them, we found PKM2 to be particularly interesting because of its reported association with tumorigenesis and cancer progression; thus, we focused on it in our further study [21, 22]. To validate the result of mass spectrometry, Co‐IP and reciprocal Co‐IP analyses were carried out using OVCAR‐3 cell lysates, confirming the endogenous interaction between PSMD14 and PKM2 (Fig. 5B). PKM2 is a protein encoded by PKM gene; it catalyzes the conversion of phosphoenolpyruvic acid (PEP) to pyruvate, which is the final and rate‐limiting step of glycolysis [21, 22]. PKM1 is another common alternatively spliced isoform of PKM gene [21, 22]. However, no interaction was observed between PSMD14 and PKM1 (Fig. 5B). Moreover, the exogenous interaction between PKM2 and PSMD14 was detected by Co‐IP using HEK293T cell lysates with nonspecific binding excluded by negative control (Fig. 5C, Fig. S2). Immunofluorescence assay was further conducted to confirm the association between PKM2 and PSMD14. We observed that PSMD14 was indeed colocalized with PKM2, and the colocalization was partial and mainly located in the cytoplasm (Fig. 5D).

Given that PSMD14 is a DUB, we explored whether PSMD14 is able to regulate ubiquitination on PKM2. The results revealed that the ubiquitination level of PKM2 was markedly increased after downregulation of PSMD14 in OVCAR‐3 cells (Fig. 5E). Additionally, application of PSMD14 specific inhibitor OPA could also lead to the elevated ubiquitination level of PKM2 (Fig. 5F). Next, we found that the level of ubiquitination on PKM2 was significantly reduced by wild‐type PSMD14, but not by PSMD14 mutants H113Q or C120S (Fig. 5G). To exclude the possibility that the deubiquitination occurs on a binding partner of PKM2 rather than PKM2 itself, we mutated all of the K residues in PKM2 to R residues and observed that the level of ubiquitination on mutated PKM2 was unaffected by PSMD14, indicating that PSMD14 mediates deubiquitination of PKM2 (Fig. S3). To investigate which types of polyubiquitination chains on PKM2 were modulated by PSMD14, three different types of HA‐tagged ubiquitin, including K11, K48, or K63‐only, were overexpressed in HEK293T cells together with Myc‐PKM2 and Flag‐PSMD14. Co‐IP showed that HA‐K63 ubiquitin was markedly reduced by Flag‐PSMD14, while HA‐K11 or HA‐K48 ubiquitin on Myc‐PKM2 did not change significantly (Fig. 5H). Therefore, these data indicated that PSMD14 decreased K63‐linked ubiquitination on PKM2.

To further confirm the effects of PSMD14‐mediated deubiquitination on PKM2 protein in ovarian cancer, we investigated whether the expression level and stability of PKM2 is regulated by PSMD14. However, knockdown and overexpression of PSMD14 did not change the expression level of PKM2 protein in OVCAR‐3 cells (Fig. S4A). Results of cycloheximide (CHX) pulse‐chase assay demonstrated that altered PSMD14 expression did not influence the stability of PKM2 protein (Fig. S4B). Previous studies have reported that PKM2 can exist in monomers as well as oligomers, including tetrameric and dimeric forms. Different with the PKM2 tetramers, the PKM2 dimers and monomers play oncogenic functions and promote proliferation, migration, invasion, and survival of tumor cells [23]. To investigate whether PSMD14 can change the ratio of different forms of PKM2 in ovarian cancer cells, cross‐linking experiments using glutaraldehyde were performed in previously described methods [24]. Knockdown of PSMD14 was observed to induce increased level of PKM2 tetramer and decreased ratio of its dimer and monomer in OVCAR‐3 cells (Fig. 5I). Tetrameric PKM2 reduced and was replaced by oncogenic dimer and monomer after overexpression of wild‐type PSMD14, but not PSMD14 mutants H113Q or C120S (Fig. 5J). Taken together, PSMD14 diminishes the ratio of PKM2 tetramer via its DUB activity.

### 3.5 PSMD14 attenuates PK activity and induces Warburg effect

**Fig. 6:**
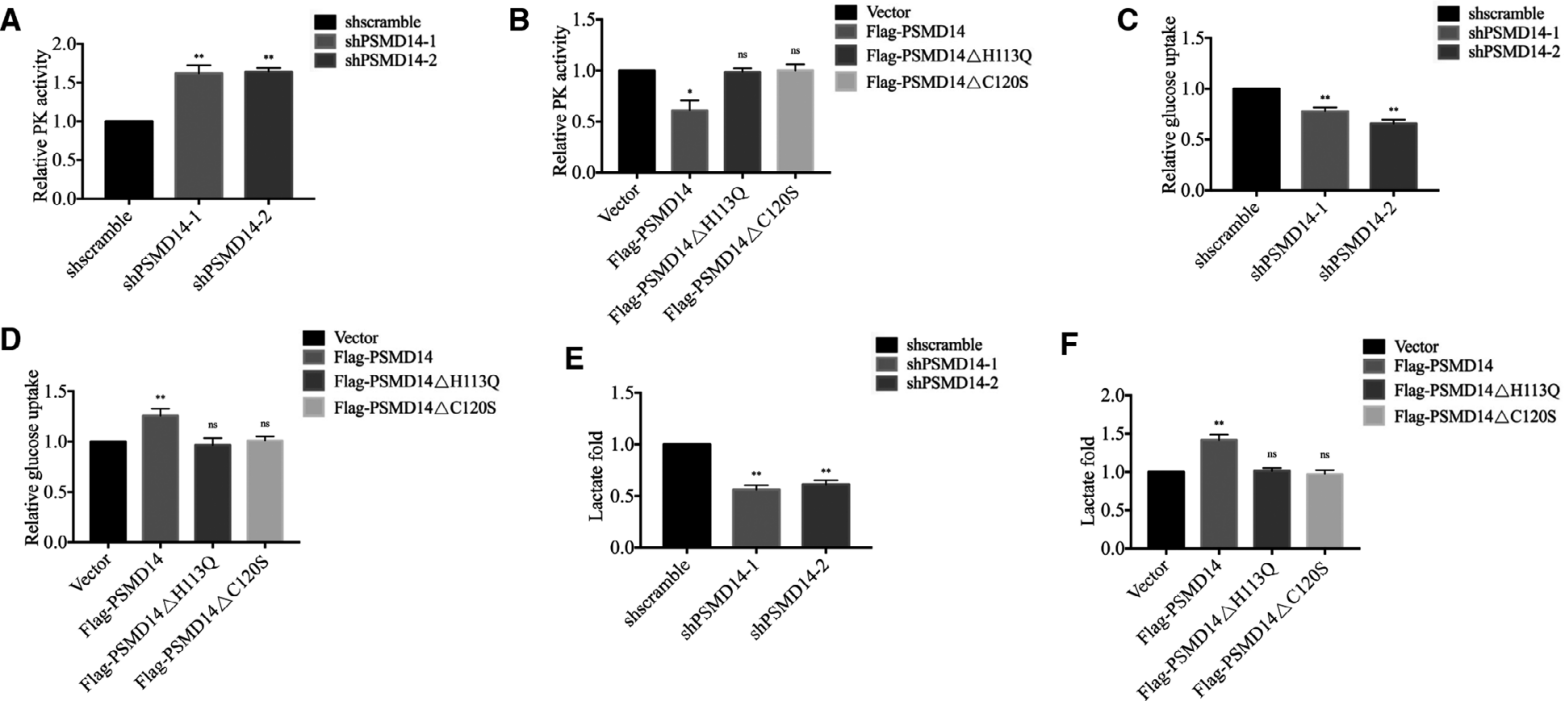
PSMD14 inhibits PK activity and increases cell glucose uptake and lactate production. (A) PK activity was detected in control and PSMD14 knockdown OVCAR‐3 cells. (B) PK activity was detected in control and PSMD14 or PSMD14 mutants overexpressed OVCAR‐3 cells. (C) Glucose uptake was measured in control and PSMD14 knockdown OVCAR‐3 cells. (D) Glucose uptake was measured in control and PSMD14 or PSMD14 mutants overexpressed OVCAR‐3 cells. (E) Lactate production was determined in control and PSMD14 knockdown OVCAR‐3 cells. (F) Lactate production was determined in control and PSMD14 or PSMD14 mutants overexpressed OVCAR‐3 cells. Error bars in this figure indicated mean ± SD. Data analyses in this figure were conducted by unpaired *t*‐test. *P < 0.05; **P < 0.01. Data represent three independent sets of experiment.

According to previous studies, PKM2 tetramer usually exists in normal cells and owns increased catalytic activity, inducing catabolism of glycolytic intermediates and production of ATP [25]. In tumor cells, PKM2 prefers to exist in dimeric or monomeric forms which have reduced catalytic activity and promote lactate production rather than normal respiratory chain, enhancing anabolic synthesis of glycolytic intermediates to support cancer cell growth [25]. Consistent with the previous results, we observed in OVCAR‐3 cells that knockdown of PSMD14 increased PK activity (Fig. 6A), whereas PSMD14 overexpression reduced PK activity significantly (Fig. 6B). We also measured glucose uptake and lactate production after silencing or overexpressing PSMD14 and found that knockdown of PSMD14 significantly reduced glucose uptake and lactate production, whereas PSMD14 overexpression caused increased glucose uptake and lactate production, which are characteristics of Warburg effect (Fig. 6C–F). However, PSMD14 mutants H113Q or C120S did not change PK activity, glucose uptake, or lactate production significantly, suggesting the necessity of DUB activity in the function of PSMD14. Therefore, our results suggested that PSMD14 contributes to metabolic reprogramming by inhibiting PK activity and inducing Warburg effect in ovarian cancer cells.

### 3.6 PSMD14 promotes the nuclear translocation of PKM2 and enhances PKM2‐dependent gene expression

**Fig. 7:**
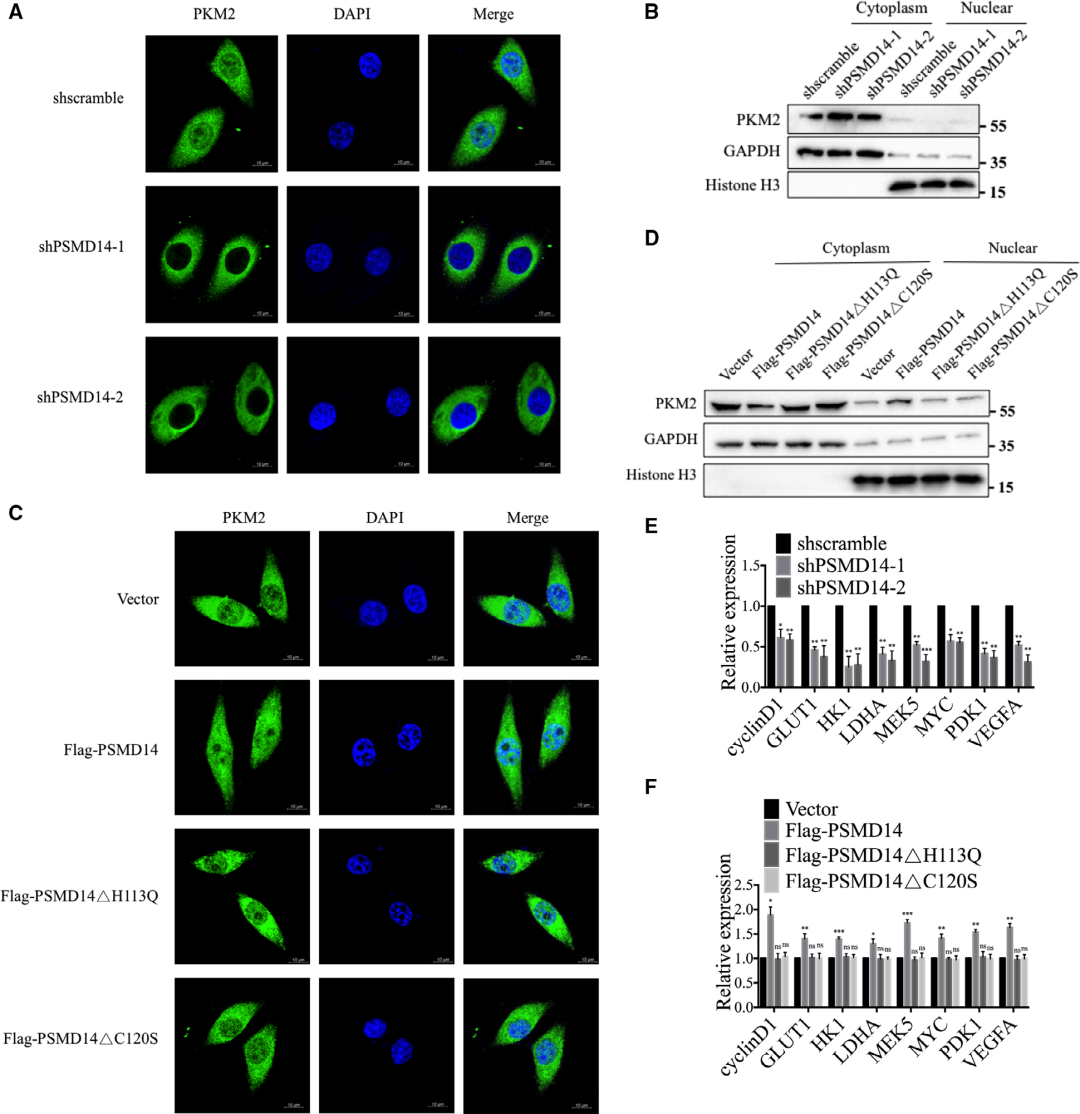
PSMD14 enhances nuclear translocation of PKM2. (A) The subcellular localization of PKM2 was determined using immunostaining with an anti‐PKM2 (green) antibody in control and PSMD14 knockdown OVCAR‐3 cells. The nucleus was stained with DAPI (blue). Scale bar, 10 μm. (B) After fractionation of the cell cytoplasm and nucleus, PKM2 was analyzed by western blotting in control and PSMD14 knockdown OVCAR‐3 cells. (C) The subcellular localization of PKM2 was determined using immunostaining with an anti‐PKM2 (green) antibody in control and PSMD14 or PSMD14 mutants overexpressed OVCAR‐3 cells. The nucleus was stained with DAPI (blue). Scale bar, 10 μm. (D) After fractionation of the cell cytoplasm and nucleus, PKM2 was analyzed by western blotting in control and PSMD14 or PSMD14 mutants overexpressed OVCAR‐3 cells. (E) The mRNA levels of genes were detected by RT‐qPCR in control and PSMD14 knockdown OVCAR‐3 cells. (F) The mRNA levels of genes were detected by RT‐qPCR in control and PSMD14 or PSMD14 mutants overexpressed OVCAR‐3 cells. Error bars in E and F indicated mean ± SD. Data analyses in E and F were conducted by unpaired *t*‐test. *P < 0.05; **P < 0.01; ***P < 0.001. Data represent three independent sets of experiment.

In addition to the functions of metabolic regulation, PKM2 can also enter the nucleus after activation of the nuclear localization signal exposed on its monomers and participate in the regulation of gene transcription [23, 25]. We then explored whether PSMD14 is associated with PKM2 nuclear translocation by both immunofluorescence staining and subcellular fractionation followed by western blot analyses. After PSMD14 was downregulated in OVCAR‐3 cells, the signal and expression level of nuclear PKM2 were decreased (Fig. 7A,B). PSMD14 overexpression enhanced PKM2 signal as well as PKM2 expression in nucleus, whereas overexpression of PSMD14 mutants H113Q or C120S could not induce these effects (Fig. 7C,D). Transcription of several genes, including cyclin D, GLUT1, HK1, LDHA, MEK5, MYC, PDK1, and VEGFA, has been reported to be enhanced by PKM2 in the nucleus via its transcriptional co‐activating functions [25, 26]. We further explored whether increased level of nuclear PKM2 induced by PSMD14 participates in transcriptional regulation of these genes. mRNA levels of these genes were found to be significantly reduced after silencing PSMD14 (Fig. 7E). Upregulation of wild‐type PSMD14, but not PSMD14 mutants, markedly promoted mRNA expression of these genes (Fig. 7F). Collectively, PSMD14 induces nuclear translocation of PKM2, promoting the expression of downstream genes associated with tumorigenesis and cancer progression.

### 3.7 PSMD14 exerts oncogenic effects through PKM2

**Fig. 8:**
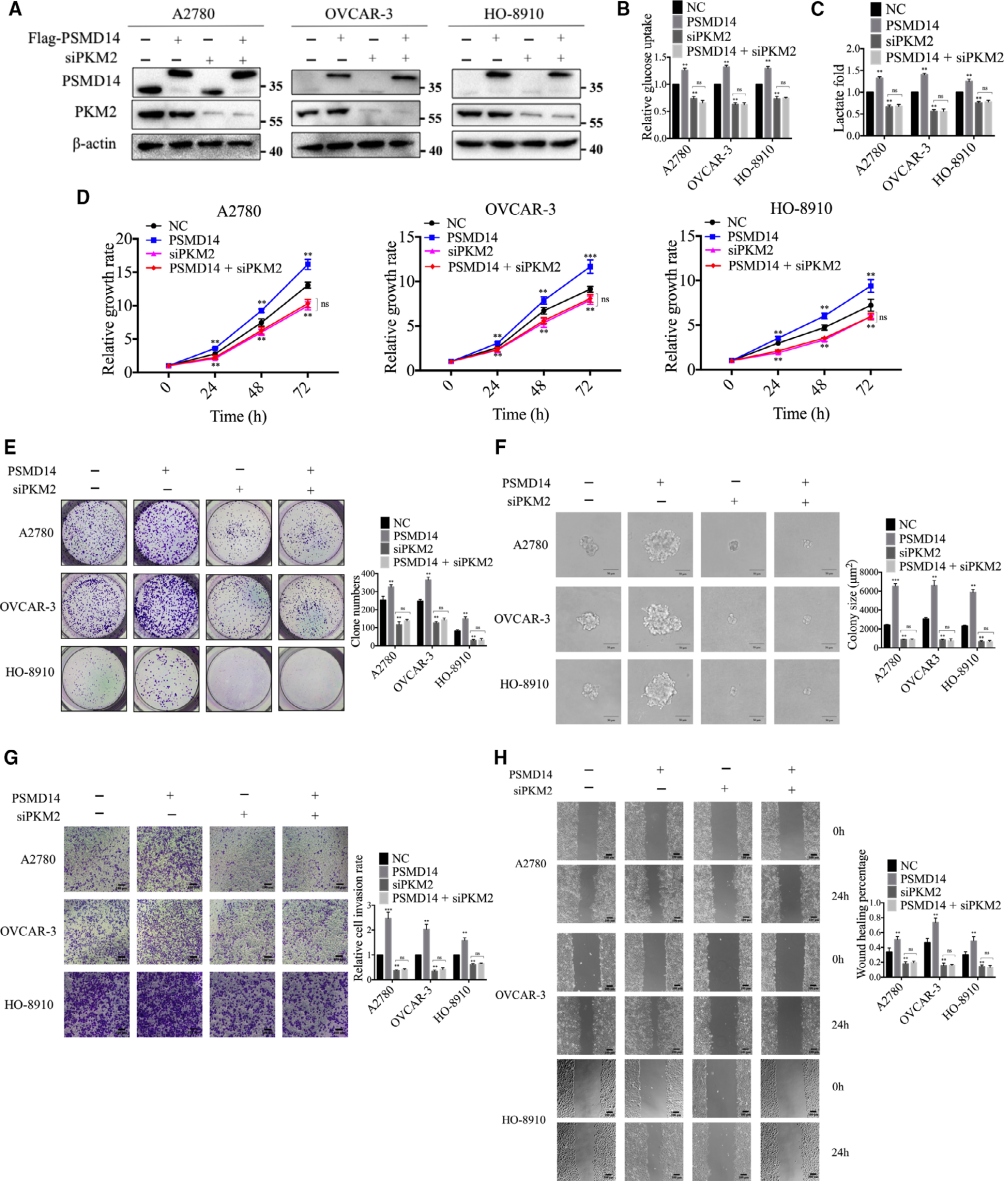
PSMD14 promotes ovarian cancer progression through PKM2. PSMD14 plasmid and anti‐PKM2 siRNA were co‐transfected into A2780, OVCAR‐3, and HO‐8910 cells; the indicated proteins (A), glucose uptake (B), lactate production (C), cell viability (D), colony formation (E), anchorage‐independent growth capacity (F), invasion (G), and migration (H) were measured. Scale bar in F: 50 μm. Scale bar in G and H: 100 μm. Error bars in B–H indicated mean ± SD. Data analyses in B–H were conducted by unpaired *t*‐test. **P < 0.01; ***P < 0.001. Data represent three independent sets of experiment.

To explore whether PSMD14 promoted malignant behavior of ovarian cancer cells through PKM2, we used RNA interference to downregulate PKM2 expression in ovarian cancer cells (Fig. 8A). As shown in Fig. 8B–H, knockdown of PKM2 inhibited glucose uptake, lactate production, viability, proliferation, invasion, and migration in ovarian cancer cells. Moreover, we inhibited the expression of PKM2 in PSMD14‐overexpressed cells. We found that silencing PKM2 abolished the induction of aerobic glycolysis and the promotion of viability, proliferation, invasion, and migration induced by PSMD14 overexpression (Fig. 8B–H). Therefore, these findings suggested that PSMD14 can function as an upstream of PKM2 to stimulate ovarian cancer progression.

**Fig. 9:**
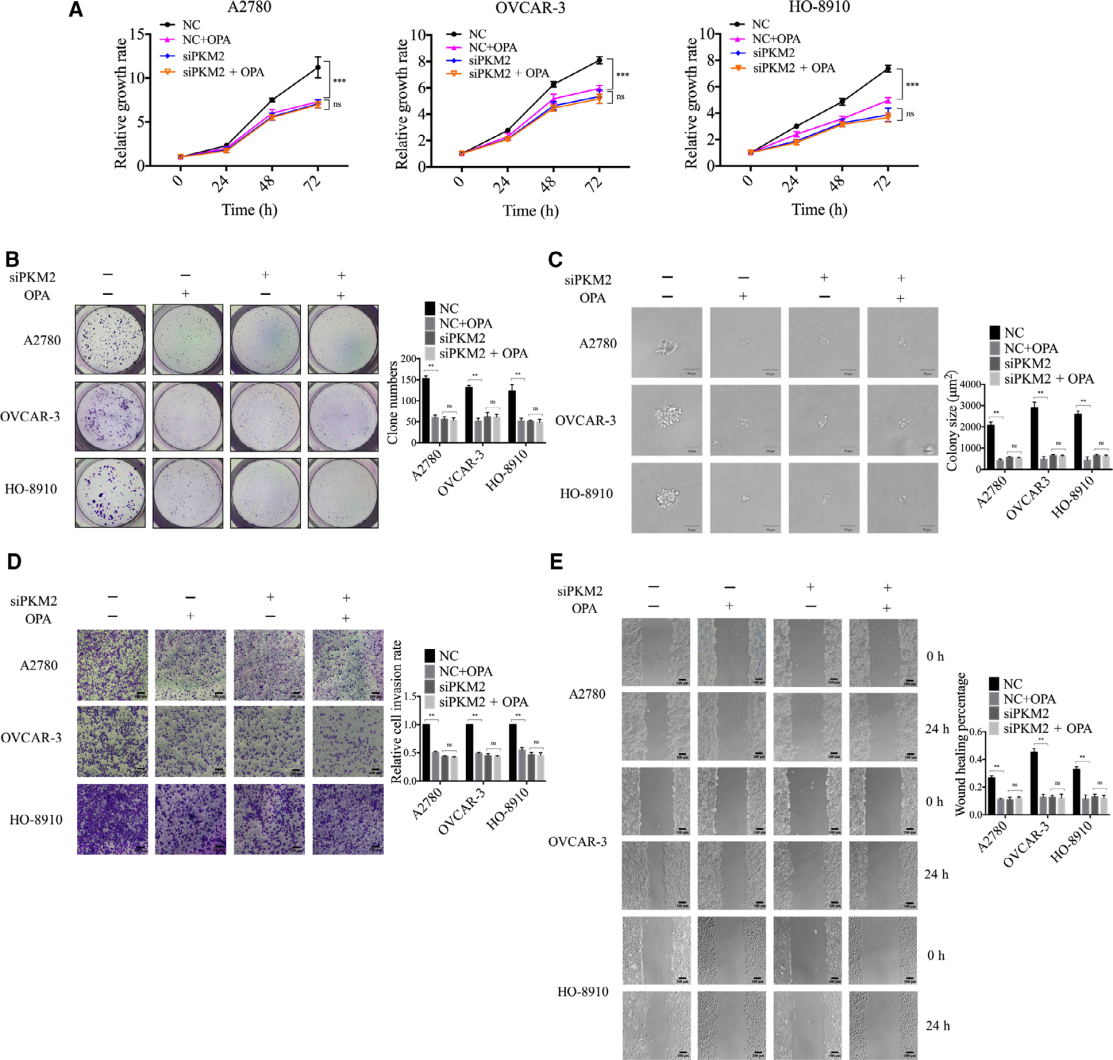
PSMD14 inhibitor O‐phenanthroline (OPA) inhibits the malignant behavior of ovarian cancer cells by preventing the ability of PSMD14 to regulate PKM2. (A) The cytotoxicity of 2.5 μm OPA to control and anti‐PKM2 siRNA transfected ovarian cancer cells was examined by CCK‐8 assay. After pretreating control and anti‐PKM2 siRNA transfected ovarian cancer cells with 2.5 μm OPA for 24 h, colony formation (B), anchorage‐independent growth capacity (C), invasion (D), and migration (E) were measured. Scale bar in C: 50 μm. Scale bar in D and E: 100 μm. Error bars in A–E indicated mean ± SD. Data analyses in A–E were conducted by unpaired *t*‐test. **P < 0.01; ***P < 0.001. Data represent three independent sets of experiment.

Additionally, to investigate whether PSMD14 inhibitor OPA impairs the malignant biological behaviors of ovarian cancer cells by preventing the ability of PSMD14 to modulate PKM2, we then applied OPA in ovarian cancer cells after downregulation of PKM2 expression. As shown in Fig. 9A–E, OPA effectively inhibited the viability, proliferation, invasion, and migration in siNC‐treated ovarian cancer cells, while had no significant effect on the malignant biological behaviors in siPKM2‐treated cells. Taken together, the antitumor effect of OPA in ovarian cancer cells is based on its inhibition of the ability of PSMD14 to decrease ubiquitination on PKM2.

## 4 Discussion

Among multiple post‐translational modification, protein ubiquitination and deubiquitination are common and important processes in cells, receiving a growing body of attention in recent years because of their validated association with various diseases, especially cancers [7]. Accruing evidence suggested that PSMD14, a member of JAMM family of DUB, exerts oncogenic functions in several human cancers, such as prostate cancer, hepatocellular carcinoma, lung adenocarcinoma, and colorectal cancer [9, 13, 14, 15]. Here, we elucidated the oncogenic roles of PSMD14 in ovarian cancer for the first time. Upregulated in ovarian cancer tissues, the level of PSMD14 expression was positively associated with FIGO stage and negatively associated with overall survival in ovarian cancer patients. PSMD14 promoted proliferation, invasion, and migration of ovarian cancer cells *in vitro* and ovarian tumor growth *in vivo*. Our results indicated that PSMD14 could be a novel gene involved in ovarian cancer progression. More importantly, PSMD14 inhibitor OPA was uncovered as a potential drug in ovarian cancer treatment.

PSMD14 can catalyze deubiquitination of various substrate molecules [10] and was disclosed to be involved in several biological processes, such as regulation of protein stability [20], transcription [18], double‐stranded DNA break repair [27], and cell cycle [16]. Some evidence suggests that PSMD14 can modulate K11‐, K48‐, and K63‐linked polyubiquitin chains in cancer [9, 17, 19]. Decreased K48‐linked ubiquitination on GRB2 induced by PSMD14 increases the stability and protein level of GRB2, promoting hepatocellular carcinoma progression [9]. PSMD14‐mediated deubiquitination of K11‐ and K63‐linked polyubiquitin chain on transcription factor E2F1 contributes to its stabilization and oncogenesis [17]. PSMD14 can inhibit lysosomal degradation and increase stability of TGF‐β receptors by removing the K63 polyubiquitin chains attached to caveolin‐1, which participates in lysosome‐dependent destruction of receptors [19]. However, specificity for K63 polyubiquitin chain was proposed to be the characteristic of JAMM family of DUB [28]. In line with this work, we observed that PSMD14 reduces only K63‐linked polyubiquitin chain on PKM2 in ovarian cancer cells, changing the forms of PKM2 oligomers without influencing the stability of PKM2 protein. *In vitro* ubiquitylation assays will be performed in the next step to confirm whether PSMD14 deubiquitinates PKM2 directly in a K63‐specific manner. Additionally, PSMD14 was also observed to interact with PKM in hepatocellular carcinoma cells according to the results of mass spectrometry carried out by Wang *et al*. [19]. Therefore, PSMD14 plays complex functions in various cancers. Whether the interaction between PSMD14 and PKM2 exists and drives tumorigenesis in other tissues warrants further investigation.

Metabolic reprogramming is considered as a hallmark of cancer, sustaining cancer cell growth and survival [29]. In recent years, ubiquitination and deubiquitination were highlighted for their potential roles in the regulation of metabolic reprogramming [7]. Our study disclosed PSMD14 as a novel DUB involved in metabolic reprogramming in ovarian cancer by interacting with PKM2. As a rate‐limiting enzyme in glycolysis, PKM2 plays a non‐negligible role in cancer cell metabolism and has been observed to participate in progression of various cancers, including ovarian cancer [30, 31]. PKM2 is encoded by PKM gene and has another spliced variant named PKM1. PKM1 exists in a high‐activity tetrameric form, whereas PKM2 can exist in dimeric or monomeric form with low activity or tetrameric form with high activity [25]. High PK activity suppresses cancer growth, while low‐activity forms of PKM2 induce Warburg effect, promoting survival of cancer cells [25]. Thereby, the conversion between different forms of PKM2 plays key roles in the regulation of its function. Molecules such as fructose 2,3‐bisphosphate (FBP) and serine have been reported to be PKM2 activators and directly involved in the allosteric regulation of PKM2 oligomers [25, 32]. PKM2 oligomers can also be altered by several post‐translational modifications, including phosphorylation, acetylation, hydroxylation, and oxidation [25]. For instance, phosphorylation on Y‐105 of PKM2 induces release of FBP, which switches PKM2 from the tetramer to the dimer [33]. Ubiquitination is another common and essential type of post‐translational modification; however, its functions in the regulation of PKM2 oligomers are still poorly known. PKM2 was reported to interact with E3 ligases including Parkin, CHIP, TRIM58, and laforin/malin complex, as well as DUBs including HAUSP and USP20 [34, 35, 36, 37, 38, 39, 40]. CHIP and HAUSP participate in the regulation of ubiquitination and stability of PKM2 protein [35, 38]. USP20 can deubiquitinate PKM2 without affecting PKM2 expression [36]. Chen *et al*. reported that Parkin modulated protein degradation of PKM2, while Liu *et al*. found that Parkin ubiquitylated PKM2 at K186 and K206 to inhibit enzymatic activity of PKM2 without affecting PKM2 stability [34, 37]. Viana *et al*. [39] observed that laforin/malin E3 ligase complex catalyzes K63‐linked ubiquitination of PKM2, which interferes with the localization of PKM2 to the nucleus. Collectively, the effect of ubiquitination and deubiquitination on PKM2 stability and activity is still controversial. Our results demonstrated that PSMD14 interacts with PKM2 and reduces K63‐linked polyubiquitin chain on PKM2, attenuating PK activity and promoting PKM2 nuclear translocation by inducing reduction in PKM2 tetramer. Previous study reported that TRIM35 interacted with PKM2, inhibiting the phosphorylation of PKM2 Y‐105 and increasing PK activity [41]. Based on this research, we speculate that PSMD14‐mediated K63‐linked deubiquitination of PKM2 perhaps affects other types of post‐translational modifications on PKM2, thereby indirectly modulating PKM2 oligomers. The exact molecular mechanism will be worthwhile to investigate in the next step.

## 5 Conclusions

In conclusion, the present study demonstrated that PSMD14 is overexpressed in ovarian cancer and promotes ovarian cancer progression by interacting with PKM2. PSMD14 can reduce K63‐linked polyubiquitin chain on PKM2 to decrease the ratio of tetrameric PKM2 without altering the expression level of PKM2 protein, thereby attenuating PK activity to induce Warburg effect and promoting PKM2 nuclear translocation to upregulate transcription of downstream cancer‐promoting genes. PSMD14 inhibitor OPA effectively inhibits malignant biological behaviors of ovarian cancer *in vitro* and ovarian tumor growth *in vivo*. These findings provided a rationale for further developing therapeutics by targeting PSMD14 in ovarian cancer.

## Conflict of interest

The authors declare no conflict of interest.

## Author contributions

TS, FB, and QY conceived the study. TS and ZL carried out the experiments. ZL analyzed the data. TS wrote the first version of the manuscript. FB and QY revised the manuscript. All of the authors approved the final version of the manuscript.

## Supporting information


**Fig. S1**. PSMD14 inhibitor O‐phenanthroline (OPA) inhibits the malignant behavior of ovarian cancer cells by specifically inhibiting PSMD14.
**Fig. S2**. Non‐specific binding in exogenous co‐IP is excluded by negative control.
**Fig. S3**. PSMD14 has no effect on the ubiquitination level of PKM2 protein with all of the K residues in which were mutated to R.
**Fig. S4**. PSMD14 has no effect on the expression level or stability of PKM2 protein.

**Table S1**. The sequences for siRNAs and primers.

**Table S2**. The proteins interacted with PSMD14 were identified by combining co‐IP and mass spectrometry.

## Data Availability

All data required to evaluate the conclusions of the paper are present in the main text or the Supplementary Materials of the paper.
