# Supplementary material for: Deubiquitinase PSMD14 promotes ovarian cancer progression by decreasing enzymatic activity of PKM2
Source: Mol Oncol. 2021 Aug 25;15(12):3639–58. doi: 10.1002/1878-0261.13076 (PMC8637564; doi:10.1002/1878-0261.13076)
Supplement: Supplementary file 1 — Fig. S1. PSMD14 inhibitor O‐phenanthroline (OPA) inhibits the malignant behavior of ovarian cancer cells by specifically inhibiting PSMD14. Fig. S2. Non‐specific binding in exogenous co‐IP is excluded by negative control. Fig. S3. PSMD14 has no effect on the ubiquitination level of PKM2 protein with all of the K residues in which were mutated to R. Fig. S4. PSMD14 has no effect on the expression level or stability of PKM2 protein. [file MOL2-15-3639-s003.docx]

**Supplementary figures**

**
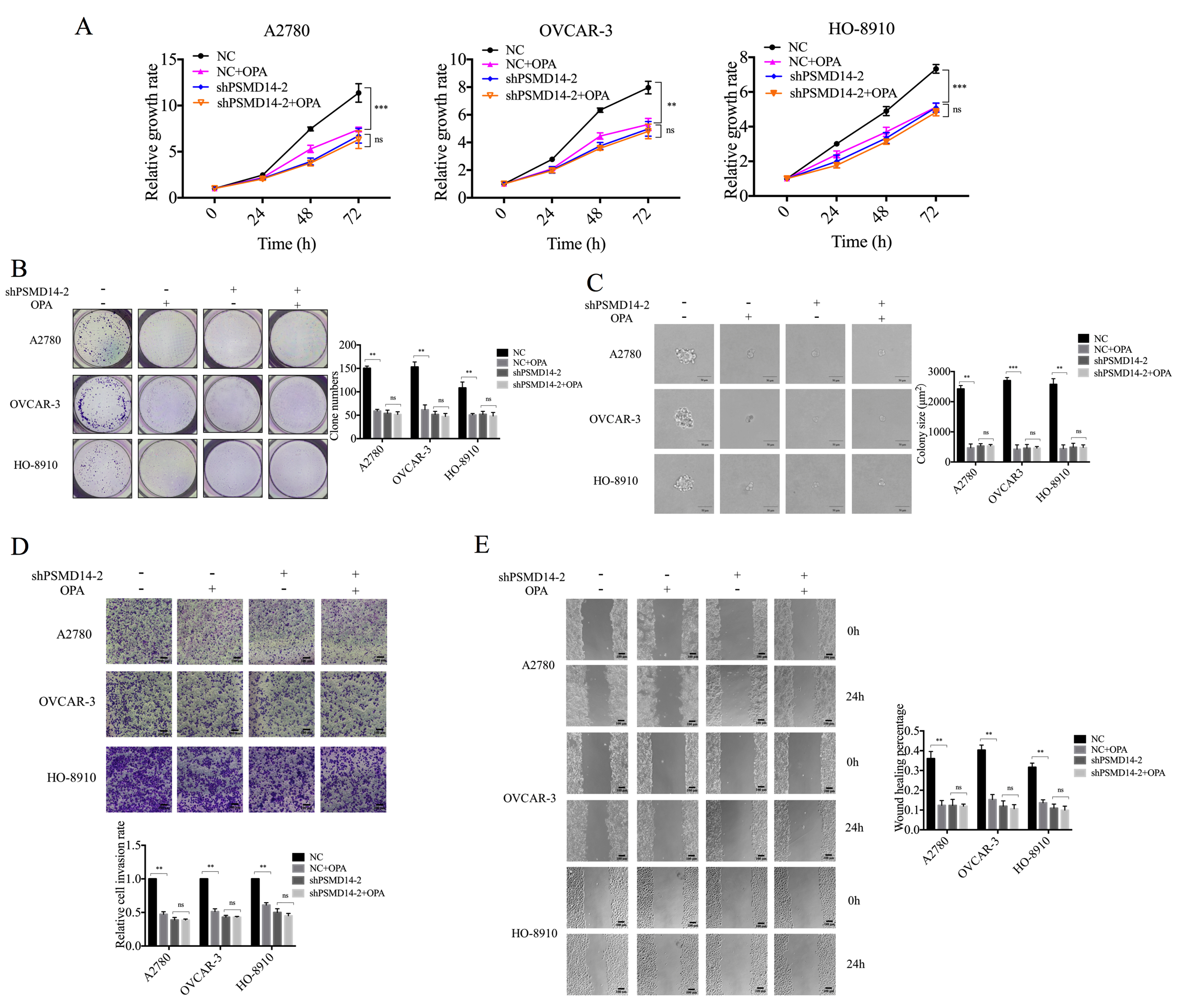
**

**Fig. S1. PSMD14 inhibitor O-phenanthroline (OPA) inhibits the malignant behavior of ovarian cancer cells by specifically inhibiting PSMD14.** (A) The cytotoxicity of 2.5 μM OPA to control and PSMD14 knockdown ovarian cancer cells was examined by CCK-8 assay. After pre-treated control and PSMD14 knockdown ovarian cancer cells with 2.5 μM OPA for 24 h, colony formation (B), anchorage independent growth capacity (C), invasion (D), migration (E) were measured. Scale bar in C: 50μm. Scale bar in D and E: 100 μm. Error bars in A, B, C, D and E indicated mean ± SD. Data analysis in A, B, C, D and E was conducted by unpaired t-test. **, P < 0.01; ***, P < 0.001. Data represent three independent sets of experiment.


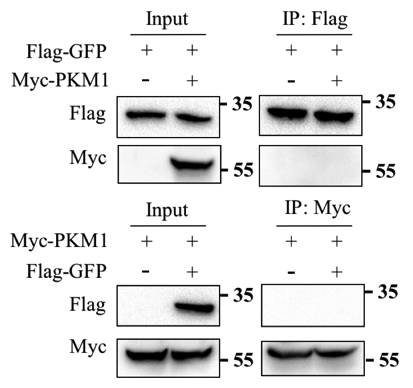


**Fig. S2. Non-specific binding in exogenous co-IP is excluded by negative control.** The Flag-GFP and Myc-PKM1 plasmids were co-transfected into HEK293T cells. Cell lysates were then immunoprecipitated by the anti-Flag or anti-Myc antibodies, respectively, and analyzed by immunoblotting.


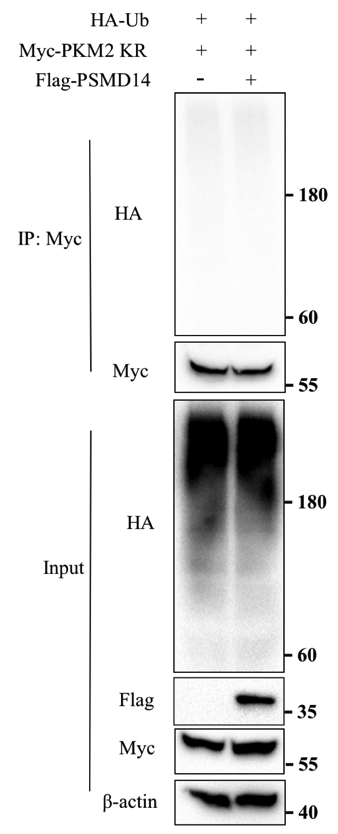


**Fig. S3. PSMD14 has no effect on the ubiquitination level of PKM2 protein with all of the K residues in which were mutated to R.** Cell lysates of HEK293T cells overexpressing Flag-PSMD14, Myc-PKM2 with all of the K residues mutated to R, and HA-ubiquitin were immunoprecipitated with anti-Myc antibody, and then detected by Western blot analysis with anti-HA and anti-Myc antibodies.


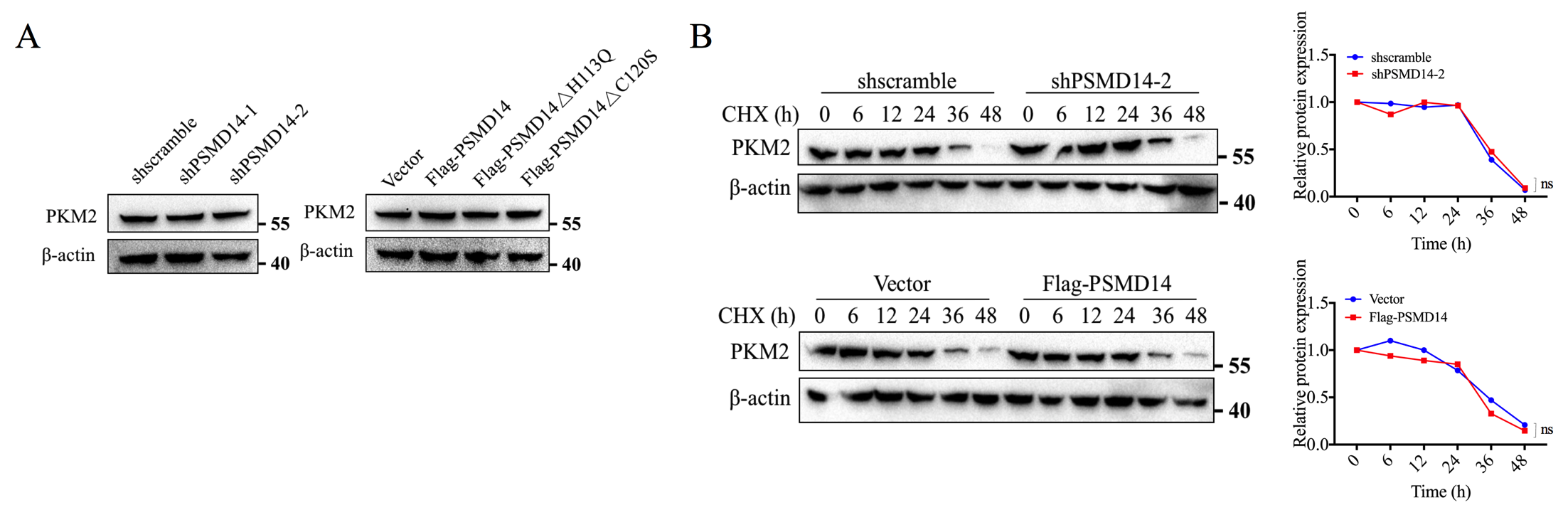


**Fig. S4. PSMD14 has no effect on the expression level or stability of PKM2 protein.** (A) Expression level of PKM2 protein was analyzed by Western blot in control and PSMD14 knockdown OVCAR-3 cells or control and PSMD14 or PSMD14 mutants overexpressed OVCAR-3 cells, respectively. (B) Control and PSMD14 knockdown or control and PSMD14 overexpressed OVCAR-3 cells were treated with cycloheximide (CHX, 100 μg/mL) for the indicated time points. The cell lysates were subjected to Western blot analyses. Data represent at least three independent sets of experiment.
