# Supplementary material for: Deubiquitinase PSMD14 promotes ovarian cancer progression by decreasing enzymatic activity of PKM2
Source: Mol Oncol. 2021 Aug 25;15(12):3639–58. doi: 10.1002/1878-0261.13076 (PMC8637564; doi:10.1002/1878-0261.13076)
Supplement: Supplementary file 2 — Table S1. The sequences for siRNAs and primers. [file MOL2-15-3639-s001.pdf]

The sequences for siRNA

PKM2	Forward, GCCAUCUACCACUUGCAAUTT	Reverse, AUUGCAAGUGGUAGAUGGCTT

The sequences for primer

MYC	Forward, GGAGGCTATTCTGCCCATTTG	Reverse, CGAGGTCATAGTTCCTGTTGGTG
LDH	Forward, CATGGCCTGTGCCATCAGTATC	Reverse, TGCCAGAGACAATCTTTGGTGTTC
GLUT1	Forward, TGTGGGCATGTGCTTCCAGTA	Reverse, CGGCCTTTAGTCTCAGGAACTTTG
cyclinD1	Forward, GCTGCGAAGTGGAAACCATC	Reverse, CCTCCTTCTGCACACATTTGAA
HK1	Forward, GCTCTCCGATGAAACTCTCATAG	Reverse, GGACCTTACGAATGTTGGCAA
MEK5	Forward, ACAGCAGCCCAGCAGTCTCA	Reverse, GTCCCGATATCGTATGTCTTGTTCA
VEGFA	Forward, GCCTTGCCTTGCTGCTCTACC	Reverse, CTTCGTGATGATTCTGCCCTCCTC
PDK1	Forward, CTGTGATACGGATCAGAAACCG	Reverse, TCCACCAAACAATAAAGAGTGCT
